# Poly (ADP-ribose) polymerase inhibitor exposure reduces ovarian reserve followed by dysfunction in granulosa cells

**DOI:** 10.1038/s41598-020-74087-9

**Published:** 2020-10-13

**Authors:** Kentaro Nakamura, Seido Takae, Eriko Shiraishi, Kiemi Shinya, Arby Jane Igualada, Nao Suzuki

**Affiliations:** Department of Obstetrics and Gynecology, St. Marianna University School of Medicine, 2-16-1 Sugao, Miyamae-ku, Kawasaki, Kanagawa 216-8511 Japan

**Keywords:** Endocrinology, Oncology

## Abstract

The use of poly (ADP-ribose) polymerase (PARP) inhibitors is expected to increase, but their effect on fertility is still unclear. The aim of this study was to investigate the effect of PARP inhibitors on ovarian function. In an in vitro study, cultures of ovaries and granulosa cells (GCs) exposed to the PARP inhibitor olaparib were evaluated by real-time RT-PCR, histological study, and hormone assays. In an in vivo study, mice were administered olaparib orally and evaluated via in vitro fertilization (IVF), follicle count, immunohistochemical staining, and real-time RT-PCR. In vitro, the gene expression of GC markers decreased in the olaparib-treated group. Olaparib also negatively affected estradiol production and the expression of GC markers in cultured GCs, with abnormal morphology of GCs observed in the treated group. The follicle number indicated depletion of follicles due to atretic changes in the treatment group, both in vitro and in vivo. Also, olaparib reduced the number of retrieved oocytes and the fertilization rate of IVF, but they recovered after 3 weeks of cessation. Our results indicate that olaparib is toxic to ovaries.

## Introduction

The number of cancer survivors has increased due to advances in treatment. The effects of cancer treatment on fertility are well known, and fertility preservation treatment and social awareness about fertility preservation are also increasing. The American Society of Clinical Oncology (ASCO) guidelines in 2013 categorize chemotherapy and radiation as treatments that may cause gonadal damage^1,2^. However, as a matter of course, ASCO guidelines cannot explain the gonadal damage caused by all cancer drugs, because new drugs are always under investigation. One class of cancer drugs which has unknown effects on fertility is poly (ADP-ribose) polymerase (PARP) inhibitors, which are presently used for breast cancer and ovarian cancer treatment.

Olaparib is a first-in-class oral PARP inhibitor used to treat patients with *BRCA* mutations that often cause breast cancer in young women, known as hereditary breast and ovarian cancer (HBOC)^3^, and patients with HER2-negative inoperable breast cancer who were previously treated with chemotherapy or who have recurrent breast cancer. Cancers with DNA repair defects, such as those with a *BRCA1* or *BRCA2* (*BRCA1/2*) mutation, can be treated with PARP inhibitors, which render them deficient in homologous recombination repair^4–6^. In tumors that lack this type of repair, PARP inhibitors block an alternative DNA repair pathway that is required for viability, resulting in their death^7^. As mentioned by the National Comprehensive Cancer Network, olaparib is also a standard maintenance therapy for patients with recurrent ovarian cancer after treatment with platinum-based chemotherapy, and for patients with or without *BRCA* mutations^8–15^. Moreover, in patients with advanced ovarian cancer with a *BRCA* mutation, olaparib is approved as a fourth-line or later treatment whether or not the patient is sensitive to platinum-based drugs^16^. Several studies have shown that olaparib as a maintenance therapy is beneficial in terms of progression-free survival in patients with newly diagnosed advanced ovarian cancer and a *BRCA1/2* mutation; such treatment provides a 70% lower risk of disease progression or death compared to placebo^16^. Other studies have also suggested that PARP inhibitors may be effective for various other cancers^17–20^. Hence, PARP inhibitors are promising drugs that are likely to be used for more patients in the future.

In addition to their effects on DNA repair, PARP inhibitors also have anti-angiogenesis effects due to their inhibition of vascular endothelial growth factor (VEGF) action^21–23^. VEGF has effects on ovarian follicular development such as primordial follicle survival, mitogenic effects on granulosa cells (GCs), and the transition from primary to secondary follicles^24^. A follicle is a dynamic unit consisting of GCs, theca cells, and an oocyte that grows from the primordial follicle for around 200 days until ovulation^25^. Follicle function is not only required for reproductive function, but also has an important function in hormone replacement^26^. Hormones are mainly produced by GCs and theca cells and are determined by gonadotropins according to the two-cell two-gonadotrophin theory^27^. GCs are critical for the follicular development process, and the follicle stage is defined by the thickness of the GC layer^26^. VEGF affects both the survival of follicles (oocytes) and the division of GCs, and is a survival factor for GCs, preventing apoptosis (ovarian follicular atresia)^28^. VEGFR-2 activity is required to enhance gonadotropin-dependent proliferation of GCs^29^.

Therefore, we hypothesized that PARP inhibitors have adverse effects on follicle development and survival due to VEGF suppression. In the present study, we investigated the ovarian toxic effects of olaparib (a PARP inhibitor) to meet the needs of fertility preservation for child-bearing patients who will receive olaparib treatment, which may become more common.

## Results

### In vitro treatment with a PARP inhibitor changes gene expression

**Figure 1:**
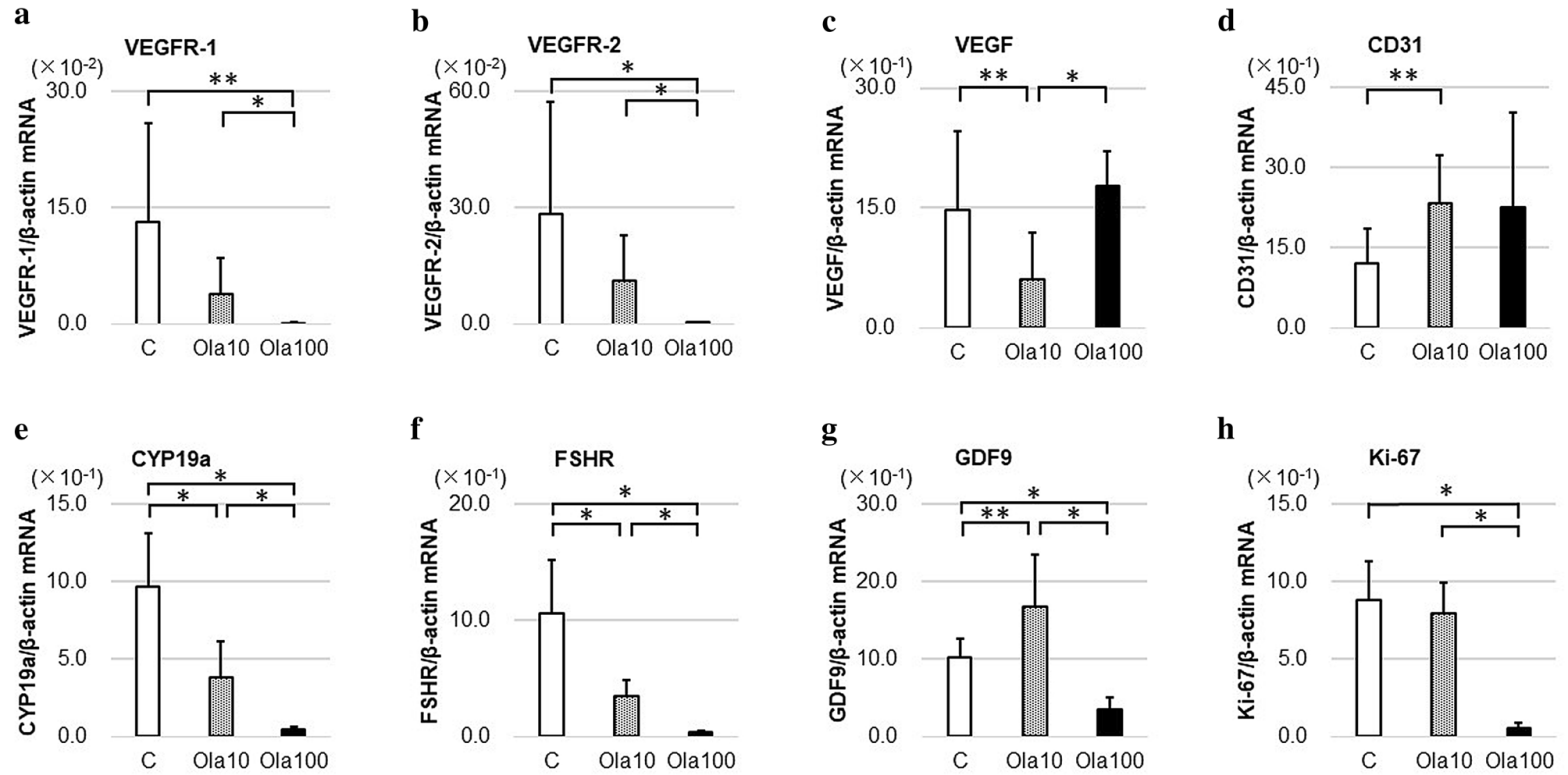
Gene expression related to ovarian reserve in vitro. Three ovaries for each group from mice at 10–11 days of age were cultured with or without olaparib (10–100 μg/ml) for 6 days, with medium changes every day before extraction of RNA and real-time RT-PCR analyses of transcript levels for various ovarian genes. (**a**) VEGFR-1 (C vs. Ola100: *p* = 0.011, Ola10 vs. Ola100: *p* = 0.0039), (**b**) VEGFR-2 (C vs. Ola100: *p* = 0.0008, Ola10 vs. Ola100: *p* = 0.0011), (**c**) VEGF (C vs. Ola10: *p* = 0.045, Ola10 vs. Ola100: *p* = 0.0049), (**d**) CD31 (C vs. Ola10: *p* = 0.025), (**e**) CYP19a (C vs. Ola10: *p* = 0.0022, Ola100 vs. others: *p* = 0.0005), (**f**) FSHR (C vs. Ola10: *p* = 0.0038, Ola100 vs. others: *p* = 0.0008), (**g**) GDF9 (C vs. Ola10: *p* = 0.037, Ola100 vs. others: *p* = 0.0005), (**h**) Ki-67 (Ola100 vs. others: *p* = 0.0005). *VEGFR* vascular endothelial growth factor receptor, *VEGF* vascular endothelial growth factor, *FSHR* follicle-stimulating hormone receptor, *GDF9* growth differentiation factor-9, *C* control group, *Ola10* olaparib 10 µg/ml group, *Ola100* olaparib 100 µg/ml group. Means ± SD of 10 samples each. *p < 0.01, **p < 0.05, significantly different between groups.

Ovaries cultured for 6 days were evaluated with real-time PCR. As shown in Fig. 1, in the olaparib group, gene expressions of GC markers *CYP19a* (C vs. Ola10: *p* = 0.0022, C vs. Ola100: *p* = 0.0005) and *FSH receptor (FSHR)* (C vs. Ola10: *p* = 0.0038, C vs. Ola100: *p* = 0.0008), as well as *Ki-67* (C vs. Ola100: *p* = 0.0005), were significantly decreased compared with the control group. The oocyte marker *growth differentiation factor-9 (GDF9)* increased in Ola10, but decreased in Ola100, showing an unclear trend. Although *VEGF* and *CD31* expressions were not changed in a concentration-dependent manner, *VEGFR type1* (C vs. Ola100: *p* = 0.011, Ola10 vs. Ola100: *p* = 0.0039) and *type2* (C vs. Ola100: *p* = 0.0008, Ola10 vs. Ola100: *p* = 0.0011) expressions were decreased in the olaparib group compared with the control group in a concentration-dependent manner.

### Changes in follicle development by the PARP inhibitor in vitro

**Figure 2:**
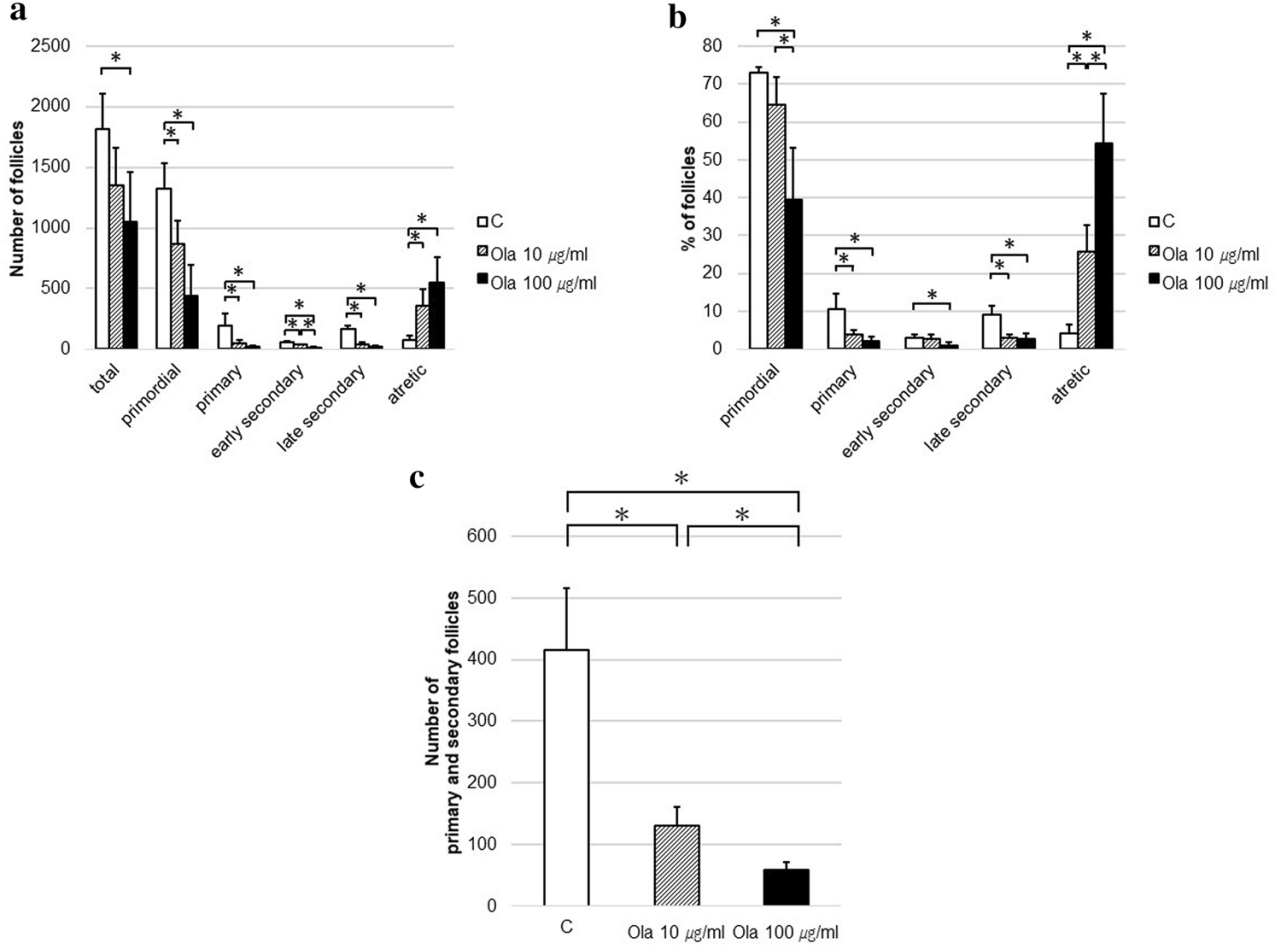
Follicle dynamics with or without treatment with olaparib in vitro. Three ovaries for each group from mice at 10–11 days of age were cultured with or without olaparib (10 or 100 μg/ml) for 8 days to evaluate follicle number count, with medium changes every 2 days. Follicle dynamics were determined at the end of the culture. (**a**) Number of follicles (total, C vs. Ola100: *p* = 0.033; primordial, C vs. others: *p* = 0.033; primary, C vs. others: *p* = 0.033; early secondary, C vs. Ola10: *p* = 0.041, C vs. Ola100: *p* = 0.032, Ola10 vs. Ola100: *p* = 0.031; late secondary, C vs. others: *p* = 0.033; atretic, C vs. others: *p* = 0.033). (**b**) Distribution of follicles (%) (primordial, Ola100 vs. others: *p* = 0.033; primary, C vs. others: *p* = 0.033; early secondary, C vs. Ola100: *p* = 0.033; late secondary, C vs. others: *p* = 0.033; atretic, *p* = 0.033). (**c**) Number of primary and secondary follicles (*p* = 0.033). *C* control group, *Ola10* olaparib 10 µg/ml group, *Ola100* Olaparib 100 µg/ml group. Means ± SD of 5 samples each. *p < 0.05, significantly different from the control group.

Cultured ovaries were histologically evaluated with HE staining and immunohistochemical staining. The follicle counts are shown in Fig. 2a. Olaparib significantly reduced the total (C vs. ola100: *p* = 0.033), primordial (C vs. others: *p* = 0.033), primary (C vs. others: *p* = 0.033), early secondary (C vs. Ola10: *p* = 0.041, C vs. Ola100: *p* = 0.032, Ola10 vs. Ola100: *p* = 0.031), and late secondary (C vs. others: *p* = 0.033) follicles and augmented the number of atretic follicles (C vs. others: *p* = 0.033). The number of follicles with GCs (primary and secondary follicles) decreased depending on the amount of olaparib (*p* = 0.033; Fig. 2c). In terms of the distribution of follicles (Fig. 2b), the percentages of primordial (Ola100 vs. others: *p* = 0.033), primary (C vs. others: *p* = 0.033), early secondary (C vs. Ola100: *p* = 0.033), and late secondary (C vs. others: *p* = 0.033) follicles significantly decreased in the olaparib group, and the percentage of atretic follicles was prominently higher than in the control group (*p* = 0.033).

### Histological evaluation of the effects of the PARP inhibitor on ovaries

**Figure 3:**
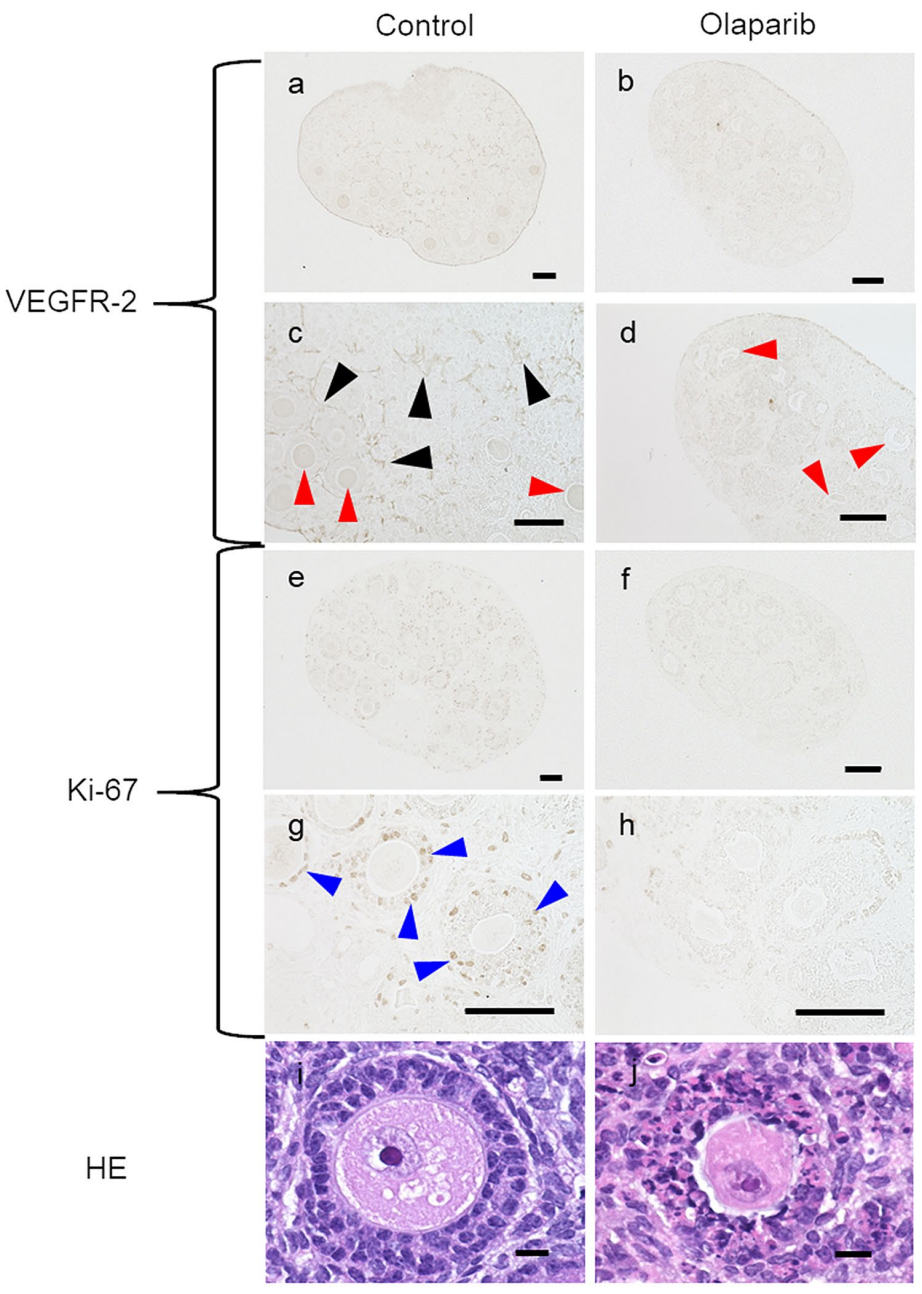
Histology evaluation of in vitro cultured ovaries with olaparib treatment. In the control group, VEGFR-2 was stained in blood vessel-like structures throughout the ovary, including around the follicle (black arrows). Also oocytes (red arrows) were stained for VEGFR-2. In the control, granulosa cells were stained for Ki-67 (blue arrows). However, granulosa cells were not stained in the olaparib group for VEGFR or Ki-67. Also, VEGFR was not expressed on oocytes of the olaparib group. HE staining shows that granulosa cells grown in olaparib did not maintain their shape and had obvious abnormalities. (**a**–**h**), scale bar = 100 µm; (**i**, **j**), scale bar = 10 µm (Color figure online).

As shown in Fig. 3, the shape of the GCs was not maintained in the olaparib group, and obvious abnormalities were observed via HE staining. VEGFR-2 (which is moderately present on oocytes^30^) and Ki-67 (a cell proliferation marker) were detected by immunohistochemical staining. In the control group, VEGFR-2 was stained in blood vessel-like structures throughout the ovaries, whereas in the olaparib groups, VEGFR-2 was not present in the blood vessel-like structures. Also, oocytes had a weak reaction for VEGFR-2 in the control group. In the control condition, Ki-67 was strongly stained, mainly in the GCs, whereas in the olaparib groups, it was not stained.

### Effects of the PARP inhibitor on granulosa cell culture

**Figure 4:**
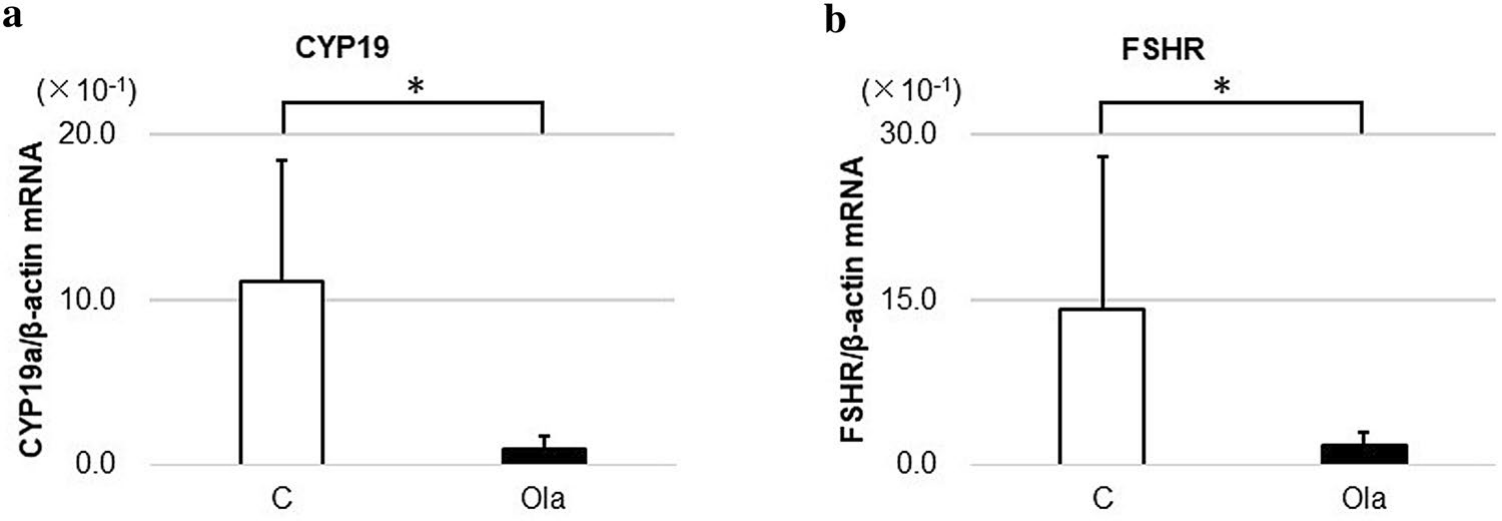
Gene expression in granulosa cell culture. Granulosa cells of three antral follicles for each group from mice at 12 weeks of age were cultured with or without olaparib for 6 h before extraction of RNA and real-time RT-PCR analyses of transcript levels. (**a**) CYP19a (*p* = 0.009). (**b**) FSHR (*p* = 0.009). *FSHR* follicle-stimulating hormone receptor, *C* control group, *Ola* olaparib group. Means ± SD of 5 samples each. *p < 0.01, significantly different between groups.

GCs were cultured for 6 h before the extraction of RNA for real-time RT-PCR analyses of transcript levels. As shown in Fig. 4, both *CYP19a* and *FSHR* showed significantly decreased gene expressions in the olaparib group (*p* = 0.009).

### Transition in the E2 level in cultured ovary media with the PARP inhibitor

**Figure 5:**
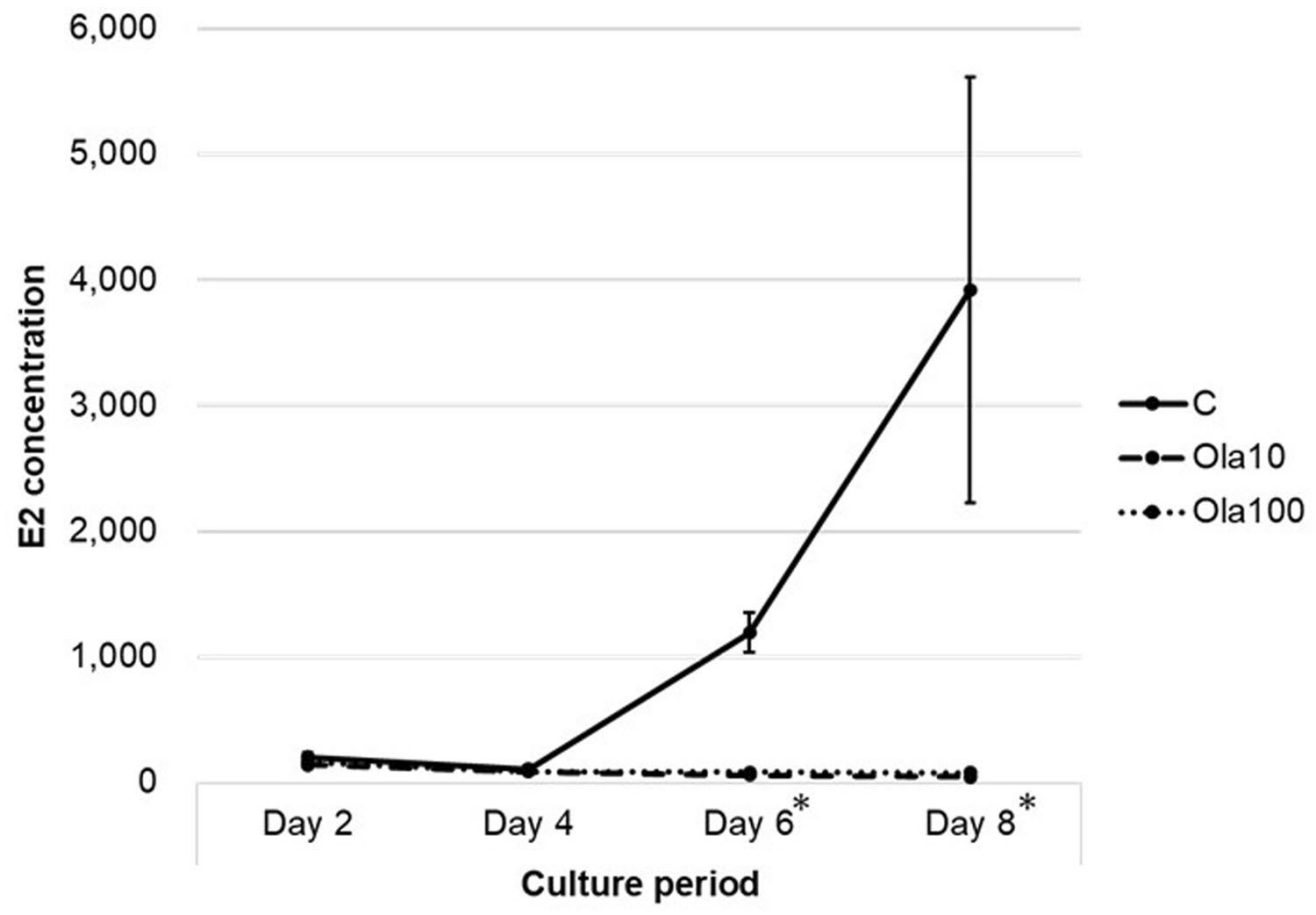
Effect of olaparib on E2 production. Individual ovaries from mice at 10–11 days of age were cultured with increasing doses (10–100 µg/ml) of olaparib with medium changes every 2 days. E2 concentrations (pg/ml) in media were measured. (Days 6 and 8, C vs. Ola10: *p* = 0.009, C vs. Ola100: *p* = 0.009). *C* control group, *Ola10* olaparib 10 µg/ml, *Ola100* olaparib 100 µg/ml group. Means ± SE of 5 samples each. *p < 0.01, significantly different from the control group.

The temporal changes in the E2 concentration are shown in Fig. 5. The E2 concentration in the control medium increased over time, but the concentration in the olaparib groups did not change. The E2 concentrations in day 8 medium were: control 3919.3 ± 1694.5, Ola10 51.6 ± 16.9, and Ola100 81.5 ± 5.4 pg/ml. The differences between the control and olaparib groups were significant (*p* = 0.009).

### Olaparib effect on in vitro fertilization

As shown in Table 1, in vitro fertilization (IVF) was compared between mice administered olaparib and vehicle to evaluate the effect of olaparib on IVF. The factors evaluated were the body weight, number of retrieved oocytes, rate of mature oocytes, fertilization rate, and arrival rate of blastocysts. The numbers of oocytes retrieved were 11.7 ± 2.2 for the control and 3.8 ± 3.0 for the olaparib group. The number of oocytes retrieved was significantly lower in the olaparib group compared to the control group (*p* = 0.0002). The fertilization rate was also lower in the olaparib group compared to the control group (*p* = 0.03).

**Table 1:** Effect of olaparib treatment and cessation on IVF.

	Control (mice: n = 10 oocytes: n = 117)	Olaparib (mice: n = 10 oocytes: n = 38)	3 weeks later	
			Control (mice: n = 10 oocytes: n = 143)	Olaparib (mice: n = 10 oocytes: n = 105)
Body weight (g)	27.8 ± 2.2^a^	26.2 ± 2.0^b^	33.3 ± 2.2^a^	33.9 ± 1.6^b^
Number of oocytes retrieved	11.7 ± 2.2^c^	3.8 ± 3.0^c,d^	14.3 ± 3.5	10.5 ± 5.3^d^
Mature oocyte rate (%)	98.3 ± 3.7	95.0 ± 9.5	96.8 ± 5.2	95.9 ± 7.5
Fertilization rate (%)	96.0 ± 6.1^e^	72.9 ± 19.6^e,f^	90.7 ± 6.8	92.9 ± 9.4^f^
Blastocyst development rate (%)	94.3 ± 9.5	97.1 ± 7.6	93.0 ± 8.0	91.2 ± 14.3

^a^*p* = 0.0005.

^b^*p* = 0.0002.

^c^*p* = 0.0002.

^d^*p* = 0.0069.

^e^*p* = 0.03.

^f^*p* = 0.033.

In addition, as a result of IVF after 3 weeks of olaparib cessation, the number of retrieved oocytes had a tendency to be lower in the olaparib group compared to the control group (*p* = 0.094). Also, after the cessation of administration, the number of oocytes retrieved was obviously recovered in the olaparib group (*p* = 0.007).

### Gene expression related to ovarian reserve changes in olaparib-treated mice

**Figure 6:**
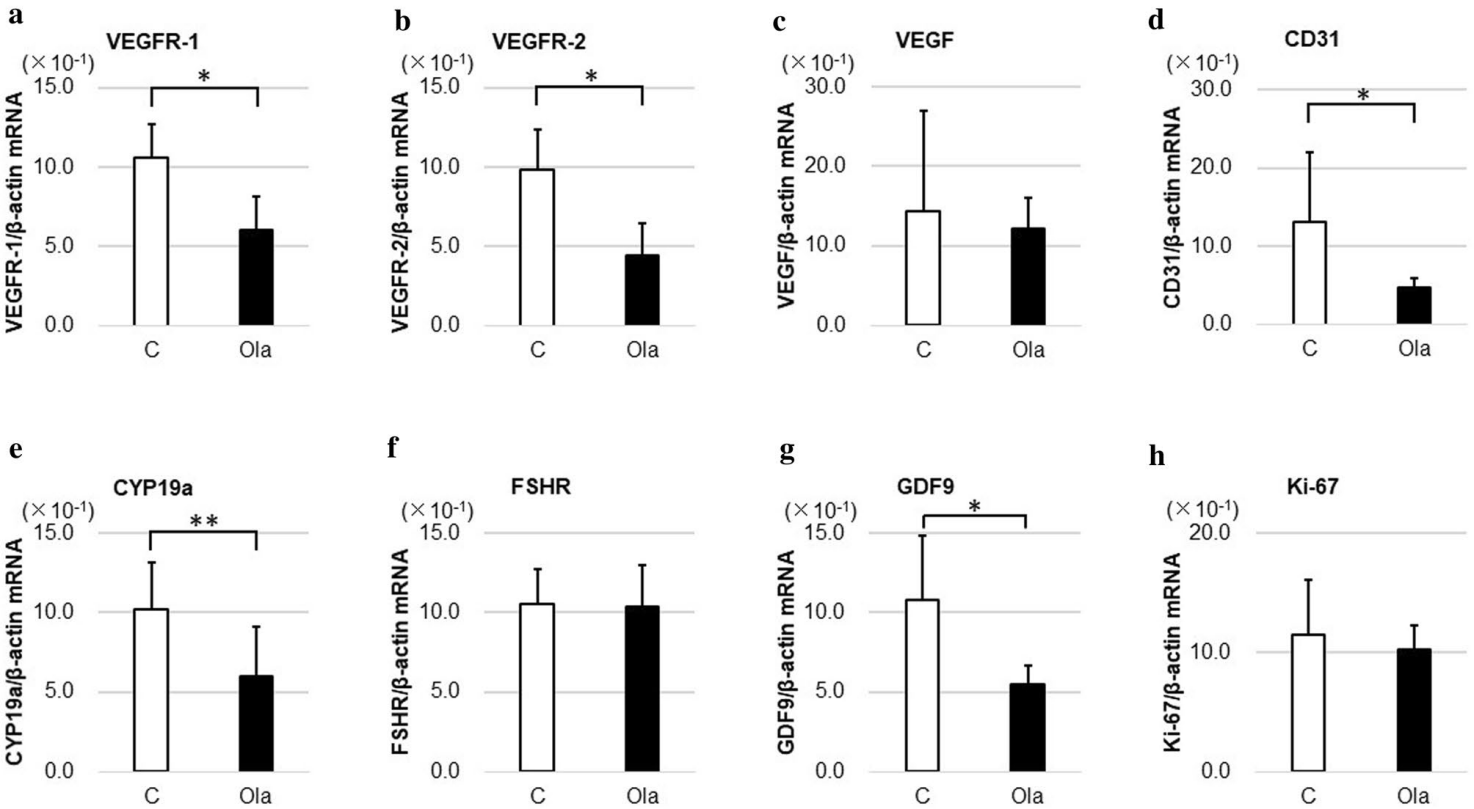
Gene expression related to ovarian reserve in vivo. Mice at 21 days of age were treated daily with control fluid (1% carboxymethyl cellulose) or olaparib (300 mg/kg body weight) for 2 weeks before real-time RT-PCR analyses of transcript levels for various ovarian genes. (**a**) VEGFR-1 (*p* = 0.0007), (**b**) VEGFR-2 (*p* = 0.0007), (**c**) VEGF, (**d**) CD31 (*p* = 0.0012), (**e**) CYP19a (*p* = 0.013), (**f**) FSHR, (**g**) GDF9 (*p* = 0.0025), (**h**) Ki-67. *VEGFR* vascular endothelial growth factor receptor, *VEGF* vascular endothelial growth factor, *FSHR* follicle-stimulating hormone receptor, *GDF9* growth differentiation factor-9. *C* control group, *Ola* olaparib group. Means ± SD of 8 control and 12 olaparib samples. *p < 0.01, **p < 0.05, significantly different between groups.

The expressions of *CYP19a*, *FSHR*, *GDF9*, *VEGF*, *VEGF receptor-1* and -*2*, *CD31*, and *Ki-67* were evaluated after 2 weeks of treatment with olaparib in vivo. As shown in Fig. 6, the gene expressions of *CYP19a* (GC marker) (*p* = 0.013), *VEGFR-1* and -*2* (angiogenesis markers, but also expressed in GCs and oocytes as growth factors) (*p* = 0.0007), *CD31* (angiogenesis marker) (*p* = 0.0012), and *GDF9* (oocyte marker) (*p* = 0.0025) significantly decreased in the olaparib group.

### Change in follicle development in olaparib-treated mice

**Figure 7:**
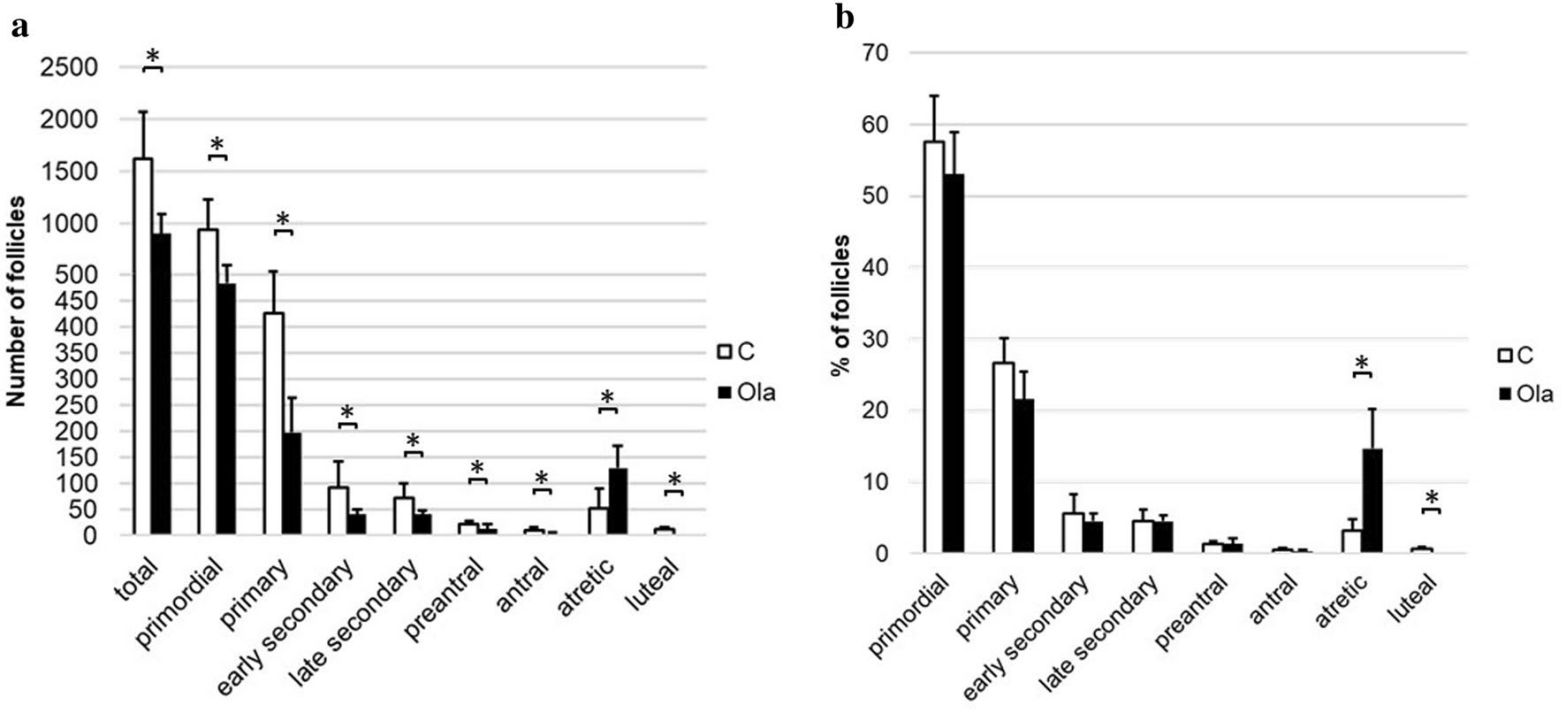
Follicle dynamics with or without treatment with olaparib in vivo. Ovaries were removed from female mice that were treated with or without olaparib for 2 weeks to evaluate the number of follicles. (**a**) Number of follicles (total, *p* = 0.028; primordial, *p* = 0.047; primary, *p* = 0.009; early secondary, *p* = 0.047; late secondary, *p* = 0.028; preantral, *p* = 0.047; antral, *p* = 0.026; atretic, *p* = 0.016; luteal; *p* = 0.0052). (**b**) Distribution of follicles (%) (primary, *p* = 0.047; atretic, *p* = 0.009; luteal; *p* = 0.0053). *C* control group, *Ola* Olaparib group. Means ± SD of 5 samples each. *p < 0.05, significantly different from the control group.

The follicle number after treatment with olaparib for 2 weeks is shown in Fig. 7. All types of developing follicles were significantly reduced in the olaparib group (total: *p* = 0.028, primordial: *p* = 0.047, primary: *p* = 0.009, early secondary: *p* = 0.047, late secondary: *p* = 0.028, preantral: *p* = 0.047, antral: *p* = 0.026). In addition, in the olaparib group, no corpus luteum was observed. Regarding the distribution of follicles, the percentage of atretic follicles was significantly higher in the olaparib group compared to the control group (*p* = 0.009).

## Discussion

The ovaries have two main reproductive functions: (1) provide oocytes for fertilization and (2) secrete sex steroid hormones (i.e., estrogen and progesterone). Therefore, female gonadotoxic agents can cause the depletion of oocytes and dysfunction in hormone production. According to previous reports, PARP inhibitors affect embryo development^31,32^. However, there is no report concerning the gonadotoxicity of PARP inhibitors in vivo. To the best of our knowledge, the present study is the first that indicates ovarian toxicity of a PARP inhibitor in vivo. PARP constitutes a large family of 18 proteins encoded by different genes and displaying a conserved catalytic domain in which PARP-1 (113 kDa), the founding member, and PARP-2 (62 kDa) are so far the sole enzymes whose catalytic activity has been shown to be immediately stimulated by DNA strand breaks^33^. They can be inhibited by olaparib, which was used in the present study.

Female fertility can be affected by cancer therapy by reducing the number of primordial follicles, compromising the hormonal balance, or inducing dysfunction of the ovaries, fallopian tubes, uterus, or cervix. Anatomic or vascular changes to these organs following surgery or radiation may also inhibit natural conception and pregnancy, leading to the need for assisted reproductive technology or a surrogate. The type of drug, dose, and protocol; the size and location of the radiation field, radiation dose, and intensity; the route of administration (oral versus intravenous); disease type; age; sex; and fertility pretreatment all impact the effect of radiation and chemotherapy^34^.

Females with cancer treated with chemotherapy and radiotherapy have a risk of premature menopause and primary ovarian insufficiency due to depletion of ovarian follicles, stromal fibrosis, and vascular damage^35^. One chemotherapy example is cyclophosphamide, which is an alkylating drug with high gonadotoxicity to dividing cells. This drug kills actively growing follicles and also activates quiescent follicles. Upon the growth of these previously quiescent follicles, they also become sensitive to the effects of cyclophosphamide^36^.

Some treatments have known effects on gonads, but the effects of other treatments are still unknown. For example, molecular targeted drugs are relatively new, and the ovarian toxicity of many of these drugs, such as imatinib, bevacizumab, and olaparib, is unknown. Although molecular targeted drugs are not considered cytotoxic, they can affect growth factors and may thus affect follicular development and ovulation. Olaparib not only has an antitumor effect, but also has anti-angiogenesis, anti-inflammatory, and immune tolerance effects^37^. Consequently, olaparib may possibly be used not only as an anticancer agent but also as a therapeutic agent for chronic inflammatory diseases and autoimmune diseases.

VEGF is important for maintaining the number of follicles^24^. The role of VEGF in ovarian folliculogenesis and steroidogenesis is consistent with the observation that follicular development and E2 synthesis are blocked by short-term treatment of monkeys with anti-VEGF antibody at the late follicular stage^38^. Furthermore, VEGFR is also required for the survival of primordial follicles^39^, and inhibition of VEGFR-2 blocks follicular development^29^. In accordance with these reports, we confirmed VEGFR expression on oocytes (Fig. 3). An increasing dose of olaparib evidently decreased the level of VEGFR. The decrease in VEGFR directly affected GC function and ovarian reserve, both in vivo and in vitro*,* as evidenced by decreased CYP19a (a GC marker), follicular count, and GDF-9 (an oocyte marker). In addition, olaparib decreased CD31 gene expression in vivo and was not observed in blood vessel-like structures via immunohistochemical staining. Therefore, it is possible that as an indirect effect, olaparib suppresses angiogenesis, reducing follicle survival and folliculogenesis. Hence, our findings are consistent with previous reports. However, the present experiments only showed a decrease in VEGFR, and downstream effects on the VEGFR pathway are still unclear. These questions should be answered by future studies.

Our in vivo experiments showed that the number of oocytes retrieved was strongly reduced. The decrease in the number of oocytes retrieved and the low E2 level could be explained by the decrease in the number of follicles. However, low gene expressions of aromatase and FSHR, as seen with the GC culture, indicated that olaparib impaired the quality and/or function of GCs. Our study demonstrated that olaparib affected both the quality and the amount of GCs. In addition, olaparib decreased the fertilization rate, but the blastocyst development rate was maintained in IVF. The factors that affect successful fertilization include oocyte nuclear and cytoplasmic maturation^40^. Furthermore, in the fertilization process, several recognizable events take place, including the sperm acrosome reaction, penetration of the egg zona pellucida by sperm, and the egg cortical reaction and zona reaction. When these processes fail, fertilization fails. VEGFR has also been identified tentatively in the zona pellucida^39^. We speculate that these reactions were interfered with by the effects on VEGFR. In addition, according to a previous report, GC promotes the capacity of oocytes to undergo fertilization^41^. Therefore, it is also possible that the fertilization rate decreased due to GC damage. However, our experiments could not determine the cause of fertilization failure. It is necessary to perform intracytoplasmic sperm injection to investigate the cause.

According to previous reports, PARP inhibition affects embryo development by clearly reducing the embryo development rate and the number of blastocysts, and by increasing the apoptotic index. The inhibition of PARylation impacts cumulus cell expansion because of abnormal changes in maturation- and expansion-related gene expression, and it decreases the embryo developmental rate and parthenogenetic activation of embryo quality^31^. Furthermore, PARylation is associated with pronuclear fusion in post-fertilization processes^32^. The result of embryo development is different between the present study and previous reports, which may be due to different study protocols.

According to the recommendation of the United States Food and Drug Administration for genotoxic pharmaceuticals after the cessation of therapy, a contraception period of 6 months, 5 times the half-life period, covers the growth and maturation phase of folliculogenesis and is expected to allow for the elimination of most damaged follicles in humans^42^. Based on this, the contraception period is approximately 3 weeks in mice, and IVF was performed 3 weeks after cessation. Comparing the olaparib group and cessation group, the number of retrieved oocytes and the fertilization rate in cessation group were significantly higher than in the olaparib group. Therefore, the effect of olaparib on IVF may be temporary. Also, we speculate that the recovery of the number of retrieved oocytes after 3 weeks of cessation was due to the elimination of the olaparib effect on cumulus cell expansion^31^. However, the number of retrieved oocytes after 3 weeks of cessation tended to decrease compared to the control group, which also had 3 weeks of cessation (*p* = 0.094). We speculate that this result may depend on the decrease of ovarian reserve due to olaparib treatment.

At present, olaparib is used for breast cancer and ovarian cancer, but it is used in ovarian cancer patients with and without *BRCA* mutations. Also, olaparib has been further expanded in the European Union for the treatment of germline *BRCA*-mutated HER2-negative advanced breast cancer. Some young patients use olaparib because those with *BRCA* mutations develop cancer at young age^43^. Although it is still controversial, *BRCA* mutations may be associated with low ovarian reserve and occult primary ovarian insufficiency^44^. Therefore, more aggressive fertility preservation treatment for patients with *BRCA* mutations may be considered.

Clinical trials for pancreatic cancer, prostate cancer, and pediatric cancer with solid tumors are being conducted, and further expansion of olaparib use is expected^45,46^. Therefore, understanding the gonadotoxicity of olaparib is important. One reason for the anti-cancer effect of olaparib is its anti-angiogenic effect, and our results are consistent with the hypothesis that this indirect effect reduces follicle survival and folliculogenesis. Based on this, the use of olaparib treatment for young cancer patients who wish to have children should be carefully considered, because the present study provides preliminary evidence of a possible risk of infertility to such patients.

**Figure 8:**
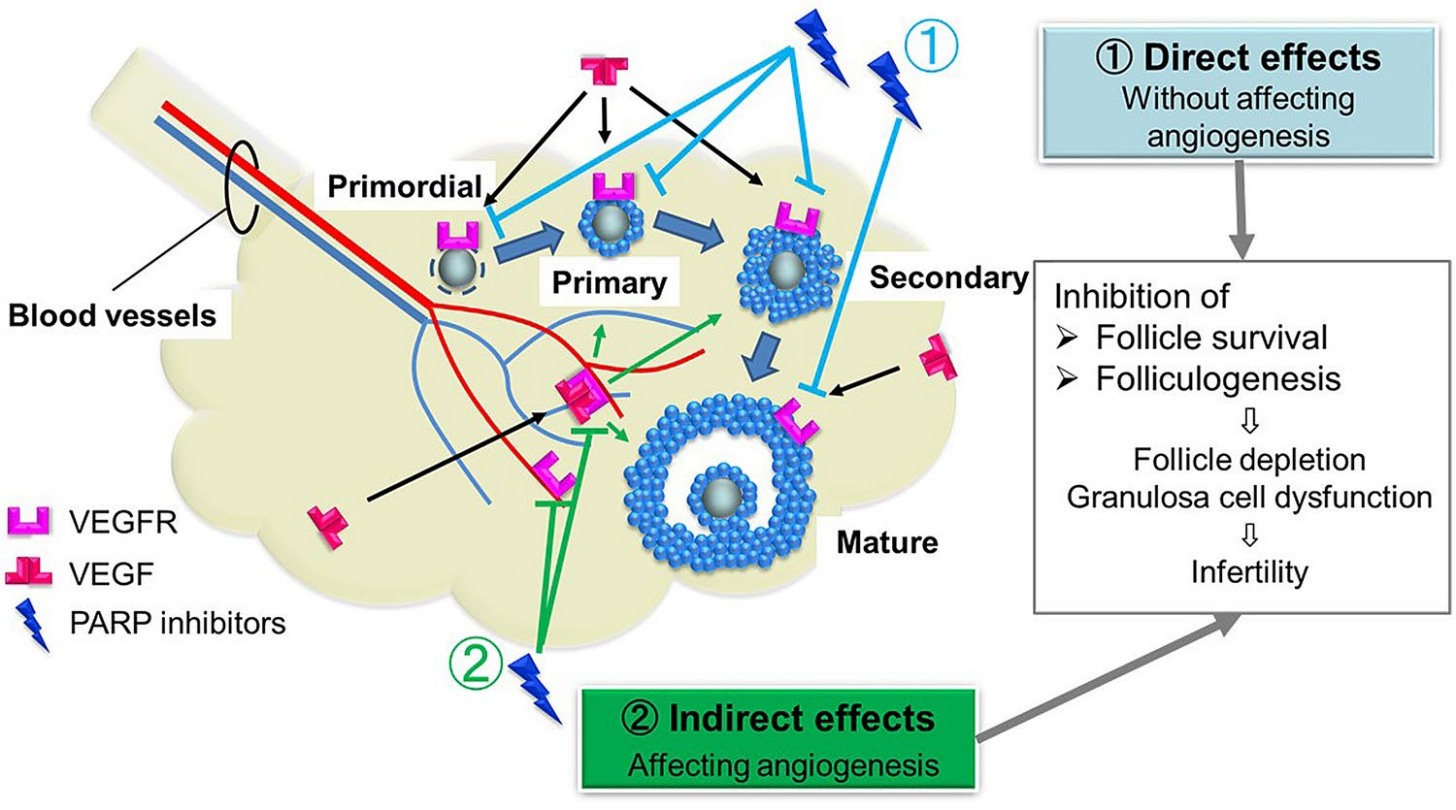
Model of olaparib gonadotoxicity. Olaparib has direct and indirect effects (with/without affecting angiogenesis) on ovarian follicles. These affect VEGFR expressed in granulosa cells, oocytes, and vessels, which may cause reduced follicle survival and folliculogenesis.

In summary, we investigated the ovarian toxicity of a PARP inhibitor, a chemotherapeutic drug that may be widely administered to younger female patients in the future. According to our investigation, olaparib has direct (without affecting angiogenesis) and indirect (affecting angiogenesis) effects on ovarian follicles that reduce follicle survival and folliculogenesis. Hence, we conclude that olaparib may lead to infertility based on low ovarian reserve and GCs dysfunction (Fig. 8).

We have shown that olaparib affects ovaries. These findings suggest the necessity for fertility preservation among young patients who will receive olaparib treatment. However, to clarify this, additional analyses using non-human primates or human clinical research are needed to extrapolate these findings, because the present study was performed with a rodent model. Also, the long-term effects of PARP inhibitors and methods to protect ovaries from damage should be studied. In addition, the effects of olaparib on the pregnancy course (pregnancy rate, live birth rate, etc.) should be examined.

## Methods

### Animals and experimental conditions

ICR mice (Japan SLC, Tokyo, Japan) were housed with a 12-h/12-h light/dark cycle in an approved animal facility, with food and water provided ad libitum. For in vitro studies, ovaries were collected from day 10–11 female mice and placed in L-15 medium (Lonza, Tokyo, Japan) supplemented with 3 mg/ml bovine serum albumin (Sigma-Aldrich, St. Louis, MO, USA). Also, ovaries were collected from mice at 12 weeks of age for the GCs culture assay. For the in vivo study (real-time PCR, IVF, and histological analysis), in brief, olaparib was mixed into 1% carboxymethyl cellulose (Wako, Tokyo, Japan) to make a 30 mg/ml suspension. Female mice were orally administered control fluid (1% carboxymethyl cellulose) or olaparib (300 mg/kg) for 2 weeks using a feeding needle, starting at postnatal day 21. The dose was determined regarding the minimum lethal dose described in the package insert. Also, as an additional study, IVF was performed to confirm the recovery from olaparib treatment after 3 weeks of cessation.

### Ethics approval

All methods were performed in accordance with the guidelines and regulations defined by the Institutional Animal Care and Use Committee (IACUC) of St. Marianna University School of Medicine. Also, all of the experimental protocols (real-time RT-PCR analyses, histological analysis, immunohistochemical analysis, and hormone assays) and animal-handling procedures (dissection, ovary culture, granulosa cell culture, and in vitro fertilization) were performed with the approval of the IACUC of St. Marianna University School of Medicine.

### Ovary culture

For the investigation of gene expression with real-time RT-PCR, 30 ovaries were placed on floating polycarbonate membranes (Merck Millipore, Tullagreen, Ireland) in 24-well plates (Greiner Bio-One, Tokyo, Japan) containing α-MEM culture medium (Thermo Fisher Scientific, Tokyo, Japan) supplemented with 3 mg/ml bovine serum albumin. Vehicle control (0.5% dimethylsulfoxide, Wako) or the PARP inhibitor (olaparib, AZD2281, Med Chem Express, Monmouth Junction, NJ, USA) at 10 or 100 µg/ml in 0.5% dimethylsulfoxide (Ola10 and Ola100, respectively) was added. Ovaries were incubated in a controlled atmosphere of 5% CO_2_ at 37 °C for 6 days, and the medium was changed every day^47^. For the hormone assay and histological study, 45 ovaries were cultured for 8 days in 0.3 IU/ml follicle-stimulating hormone (FSH), and the medium was changed every other day.

### Histological analysis

At the end of the culture, ovaries (n = 5) were fixed in 10% buffered formalin for 12 h and embedded in paraffin wax. Serial sections of 6 µm thickness were cut and stained with hematoxylin and eosin (HE) for histomorphological observation and follicle number counting. The stages of follicular development were defined as follows: primordial follicle (a single layer of flattened pre-GCs), primary follicle (one cuboidal GC layer), early secondary follicle (two cuboidal GC layers, no antrum), late secondary follicle (more than two cuboidal GC layers without an antrum cavity), preantral follicle (multiple layers of cuboidal GCs with scattered antrum cavities), and antral follicle (multiple layers of cuboidal GCs with a large single antrum cavity). Follicles with pyknotic nuclei in the GCs and/or degeneration of the nucleus of the oocyte were defined as atretic follicles. To prevent double-counting, only follicles with oocyte nuclei were counted^48–50^.

### Immunohistochemical analysis

Immunohistochemical staining was performed according to the manufacturer’s instructions. Briefly, sections were blocked in 5% skim milk in phosphate-buffered saline (PBS) (Wako) at room temperature for 10 min after antigen retrieval with heating. Immunoreactions were performed with primary antibodies in PBS at 4 °C overnight. Primary antibodies for immunohistochemistry were rabbit monoclonal anti-VEGFR-2 (Cell Signaling Technology, Danvers MA, USA; 1:100) and rabbit monoclonal anti-Ki-67 (Cell Signaling Technology; 1:400). After washing with PBS, the sections were reacted with reagents from the Histostain Plus immunohistochemistry kit (Life Technology, Carlsbad, CA, USA). The peroxidase reaction was developed using a diaminobenzidine reaction kit (Abcam, Tokyo, Japan). The sections were observed with a microscope (Bio-Revo BZ-X700, KEYENCE, Tokyo, Japan).

### Real-time RT-PCR analyses

Total RNA extraction was performed using an RNeasy Micro Kit, and cDNA synthesis was performed using a Sensicript RT Kit (QIAGEN, Hilden, Germany). Real-time PCR was performed using PowerUp SYBR Green Master Mix (Applied Biosystems, Foster City, CA, USA) on a StepOne plus real-time PCR system (Applied Biosystems) as follows: 20 s at 95 °C, and 40 cycles of 3 s at 95 °C and 30 s at 60 °C. The relative abundance of specific transcripts was normalized using β-actin as a housekeeping gene^48^. The relative expression of the mRNAs of interest was calculated with the *ΔΔ*CT method. The primers used are shown in Table 2.

**Table 2:** Primers used for RT-PCR.

Primer name	Forward (5′–3′)	Reverse (5′–3′)
β-actin	GTATCCATGAAATAAGTGGTTACAGG	GCAGTACATAATTTACACAGAAGCAAT
GDF9	TTTCCCCCAAAACGAGTGTG	TCGGGTTCAATGGTCAACAC
CYP19a	GCACAGTCACTACATCTCCCGA	CACACAAACTTCCACCATTCGA
CD31	TTAGTGTTTCGCTGCCAAGC	AGCTTCACTGCTTTGCTTGG
VEGF	GATGTGAATGCAGACCAAAG	CACATCTGCAAGTACGTTCG
FSHR	TCTAACAGGGTCTTCCTCTGC	CTCAGTTCAATGGCGTTCC
Ki-67	AGGCCCAAGTTTGATGCATC	TTCTGCAGCTGGTTTGCTTG
VEGFR-1	TTCTGTCCTCCAGAAAGTGC	ATCCATTTTAGGGGAAGTCG
VEGFR-2	ACGTTTGAGAACCTCACGTG	AGCTGTTTGACCAGGCAATG

*GDF9* growth differentiation factor 9, *VEGF* vascular endothelial growth factor, *FSHR* follicle stimulating hormone receptor, *VEGFR-1* vascular endothelial growth factor receptor type 1, *VEGFR-2* vascular endothelial growth factor receptor type 2.

### Hormone assays

17β-Estradiol (E2) concentrations in medium (n = 5) were measured using an Estradiol EIA Kit (Cayman Chemical Co., Ltd., Ann Arbor, MI, USA) following the manufacturer’s protocol. The plate was read at a wavelength of 412 nm.

### Granulosa cell culture

Antral follicles were separated from 10 ovaries for the collection of GCs. The GCs were collected by gently scraping the follicle wall with 27G needles and were pooled. After washing with DMEM, the collected GCs were cultured in plastic 24-well plates in 400 µl Ham’s F-12: Dulbecco’s minimal essential medium (1:1, v/v) (Sigma-Aldrich) containing 10% fetal bovine serum (Cosmo Bio Co., Ltd., Tokyo, Japan) and 1% antibiotic–antimycotic (Nacalai, Kyoto, Japan) with/without olaparib at 37 °C in 5% CO_2_ for 6 h. The GCs were washed with PBS after culturing for 6 h and were used for real-time RT-PCR analyses of transcript levels.

### In vitro fertilization (IVF)

Animals (n = 10) were administered olaparib or control fluid and were then treated i.p. with human chorionic gonadotropin (hCG) (10 IU/mice) (Asuka Animal Health, Tokyo, Japan) to induce ovulation when vaginal cytology indicated pro-estrus. At 16 h after treatment, the number of ovulated oocytes was determined. For IVF, sperm from ICR male mice were collected into TYH (LSI Medience Co., Tokyo, Japan) and preincubated for 30 min at 37 °C in 5% CO_2_. Sperm were transferred to a TYH culture drop containing cumulus-oocyte complexes and incubated at 37 °C in 5% CO_2_ for 6 h to facilitate fertilization. After fertilization, ova were recovered from the TYH culture drops, transferred to drops of KSOM medium (Merck Millipore, Burlington, CA, USA), and cultured at 37 °C in 5% CO_2_ in the air. After culturing for 16 h, two-cell embryos were transferred to fresh KSOM medium drops and cultured at 37 °C in 5% CO_2_ to observe development up to the blastocyst stage^51^.

### Statistics

The data are expressed as mean ± standard error (hormone assay) and as mean ± standard deviation (others). Statistical analyses were performed with the Steel–Dwass test or Mann–Whitney U test as appropriate, and a *P* value of less than 0.05 was considered statistically significant. The JMP Pro12 package program was used for statistical analyses.
